# An Identity Authentication Method Combining Liveness Detection and Face Recognition

**DOI:** 10.3390/s19214733

**Published:** 2019-10-31

**Authors:** Shuhua Liu, Yu Song, Mengyu Zhang, Jianwei Zhao, Shihao Yang, Kun Hou

**Affiliations:** School of Information Science and Technology, Northeast Normal University, Changchun 130117, China; Liush129@nenu.edu.cn (S.L.); songy590@nenu.edu.cn (Y.S.); zhangmy167@nenu.edu.cn (M.Z.); zhaojw374@nenu.edu.cn (J.Z.); yangsh861@nenu.edu.cn (S.Y.)

**Keywords:** liveness detection, Kinect camera, infrared radiation, deep learning, FaceNet

## Abstract

In this study, an advanced Kinect sensor was adopted to acquire infrared radiation (IR) images for liveness detection. The proposed liveness detection method based on infrared radiation (IR) images can deal with face spoofs. Face pictures were acquired by a Kinect camera and converted into IR images. Feature extraction and classification were carried out by a deep neural network to distinguish between real individuals and face spoofs. IR images collected by the Kinect camera have depth information. Therefore, the IR pixels from live images have an evident hierarchical structure, while those from photos or videos have no evident hierarchical feature. Accordingly, two types of IR images were learned through the deep network to realize the identification of whether images were from live individuals. In comparison with other liveness detection cross-databases, our recognition accuracy was 99.8% and better than other algorithms. FaceNet is a face recognition model, and it is robust to occlusion, blur, illumination, and steering. We combined the liveness detection and FaceNet model for identity authentication. For improving the application of the authentication approach, we proposed two improved ways to run the FaceNet model. Experimental results showed that the combination of the proposed liveness detection and improved face recognition had a good recognition effect and can be used for identity authentication.

## 1. Introduction

Face recognition is the most efficient and widely used among various biometric techniques, such as fingerprinting, iris scanning, and hand geometry. The reason is that this method is natural, nonintrusive, and low cost [1]. Therefore, researchers have developed several recognition techniques in the last decade. These techniques can generally be divided into two categories according to the face feature extracting methodology: methods that manually extract features on the basis of traditional machine learning and those that automatically acquire face features on the basis of deep learning. The accuracy of face recognition is greatly improved using the deep learning network because of its capability to extract the deep features of human faces. FaceNet is a face recognition model with high accuracy, and it is robust to occlusion, blur, illumination, and steering [2]. It directly learns a mapping from face images in a compact Euclidean space where distances directly correspond to a measure of face similarity. Once this space has been produced, tasks such as face recognition can be easily implemented using standard techniques with FaceNet embeddings as feature vectors. In addition, end-to-end training of FaceNet simplifies the setup and shows that directly optimizing a loss relevant to the task at hand improves performance. In this study, for improving the application of the FaceNet model, we proposed two improved ways, namely, by improving the model and by building “unknown” data classification. The details will be introduced in Section 3.2.

Although the improved FaceNet framework can accurately recognize human faces, like other recognition systems, it cannot prevent cheating. Most existing face recognition systems are vulnerable to spoofing attacks. A spoofing attack occurs when someone attempts to bypass a face biometric system by presenting a fake face in front of the camera. For instance, the researchers in [3] inspected the threat of the online social network-based facial disclosure against that based on some commercial face authentication systems. Common spoof attacks include photos, videos, masks, and replayed 3D face models.

Therefore, this paper proposes a liveness detection approach based on infrared radiation (IR) images acquired using a Kinect camera. IR images from live faces are used as positive samples, while IR images from photos or videos are used as negative samples. The samples above are input into the convolutional neural network (CNN) for training to distinguish live faces and spoof attacks. After liveness detection, an improved FaceNet will continue to recognize a face and provide the corresponding ID or UNKNOWN output for accurate identity authentication.

The rest of the paper is organized as follows. Section 2 briefly reviews the related works and recent liveness detection methods. Section 3 presents a framework that combines liveness detection and face recognition, and then the proposed liveness detection method based on IR image features and an improved FaceNet model, called IFaceNet, are described. Section 4 presents the experimental verifications. Section 5 elaborates the conclusions.

## 2. Related Works

Face recognition has gradually become an important encryption and decryption method because of its rapidity, effectiveness, and user friendliness. However, the security issues of face recognition technology are becoming increasingly prominent. Therefore, liveness detection has become an important part for reliable authentication systems. With the development of the Internet, criminals collect user face images from the Internet and produce fake faces to attack an authentication system. Ref. [3] passed the authentication of six commercial face recognition systems, namely, Face Unlock, Facelock Pro, Visidon, Veriface, Luxand Blink, and FastAccess, by using photos of valid users. Common spoof faces include photos (print), videos (replay), masks, and synthetic 3D face models. Among them, photos and videos are 2D fake faces that are less expensive for spoof attacks and are the two most popular forms of deception. Therefore, it is urgent to introduce liveness detection into identity authentication systems to improve the practicality and safety of face recognition. Liveness detection methods can obtain different classification systems depending on different classification criteria. According to their application forms, current mainstream liveness detection methods are divided into interactive and noninteractive categories. Interactive detection methods use action instructions to interact with users and require users to cooperate to complete certain actions. The noninteractive method does not need user interactions and automatically completes the detection task. According to the extraction methods of face features, these methods can be divided into two categories: manual feature extraction and automatic feature extraction using a deep learning network.

Common liveness detection methods are mainly based on texture, life information, different sensors, and deep features. Live faces have complex 3D structures, while photo and video attacks are 2D planar structures. Different light reflections of surfaces from the 3D and 2D structures will exhibit differences in bright and dark areas of facial colors. Texture-based methods mainly use these differences as clues to classify live and fake faces. The detection method based on texture is implemented using Local Binary Pattern(LBP) [4,5] and improved LBP [6,7] algorithms. This method has a low computational complexity and is easy to implement, but it is greatly influenced by hardware conditions. The accuracy of the algorithm decreases when the image quality is low. The method based on life features uses vital signs, such as heartbeat [8,9], blood flow [10], blinking [11,12], and involuntary micromotion of facial muscles [13,14], to classify live and fake faces. Under the constraint conditions, this method has a high detection accuracy if life features can be extracted stably; however, this method requires face video as the input and needs a large amount of computation. Even more unfortunately, the simulated micromotion of fake faces can also attack this method. The method based on different sensors adopts different image acquisition systems, such as a multispectral camera, an infrared camera, a deep camera, and a light field camera, to capture corresponding types of human face images for liveness detection. The overall recognition accuracy of this method is high, but this method needs to add new hardware and, thus, increases the system cost. The methods based on deep features involve training of the initial CNN to extract depth features followed by classification [15,16,17]. These methods use pretrained ResNet-50, VGG, and other models to extract features [18,19,20] and 3D convolution to extract spatiotemporal deep features [21,22].

Given that deep learning can extract high-level abstract features of human faces autonomously, noninteractive liveness detection using deep learning is a future development trend. With the gradual popularization of face recognition systems and the decrease in the price of hardware, it is necessary and worthy by adding image acquisition equipment into some important face authentication systems to improve the security and reliability of them. Liveness detection of faces using real depth information is not commonly used in biometrics technology and the literature [23]. All publicly available datasets such as CASIA, NUAA, and PRINT-ATTACK DB are designed for 2D spoofing prevention, and no depth data are included in these datasets. Therefore, we adopted a Kinect camera acquiring real infrared radiation (IR) images for liveness detection.

## 3. Methodology

### 3.1. Problem Statements

With the popularity of face recognition, criminals will try to attack the face recognition system, for which liveness detection has become an important part of the authentication system. Among the current liveness detection algorithms, methods based on deep learning with IR images collected by Kinect cameras are rarely reported. Therefore, we proposed this method in this paper. Infrared images were captured by a Kinect camera as the training data. The data from real faces served as positive samples, while the data from photos or videos served as negative samples for training the CNN network to distinguish whether a face is live. In addition, because the FaceNet model has a high face recognition accuracy, this paper put forward two improved ways to use FaceNet for applications. The improved FaceNet combined with a liveness detection algorithm to form an integrated authentication system.

### 3.2. Model Framework

**Figure 1:**
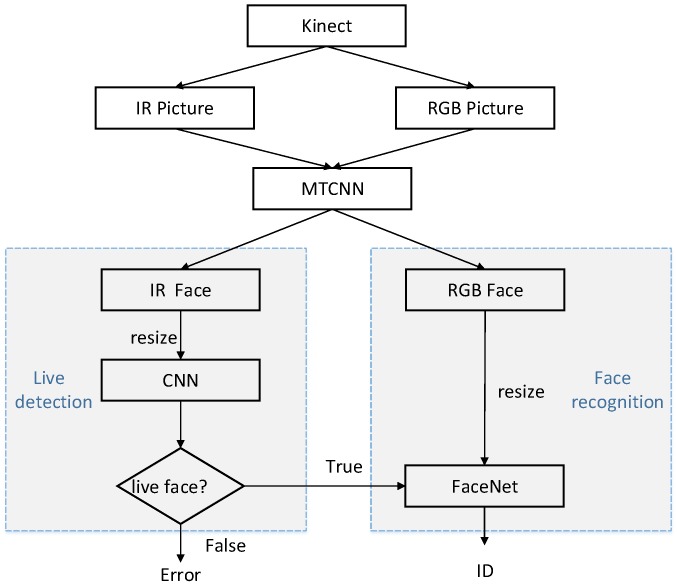
Identity authentication framework based on liveness detection and FaceNet. CNN, convolutional neural network; IR, infrared radiation; MTCNN, multitask cascaded CNN.

The proposed framework that combines FaceNet with liveness detection is shown in Figure 1.

Firstly, we adopted a Microsoft Kinect camera to collect RGB and IR images of human faces. Secondly, a multitask cascaded convolutional network (MTCNN) [24] was used to clip and align the face parts of RGB and IR images. Finally, the IR images processed by MTCNN were used to train the CNN for liveness detection, while RGB images were utilized to train the FaceNet model for face recognition. When the results of liveness detection are true, face recognition is continued to complete the entire authentication process. If the liveness detection is false, then the operation of the algorithm is terminated, and face recognition is no longer performed.

The MTCNN consisted of three stages. First, candidate windows were produced through a fast proposal network (P-Net) through a shallow CNN. Second, candidates were refined in the next stage through a refinement network (R-Net) to reject a large number of nonface windows through a more complex CNN. Third, the output network (O-Net) produced a final bounding box and facial landmark positions by using a more powerful CNN to refine the results and output the facial landmark positions.

In the following sections, the liveness detection based on IR images and the face recognition algorithm based on improved FaceNet are described in detail.

### 3.3. Liveness Detection Based on IR Image Features (LDIR)

**Figure 2:**
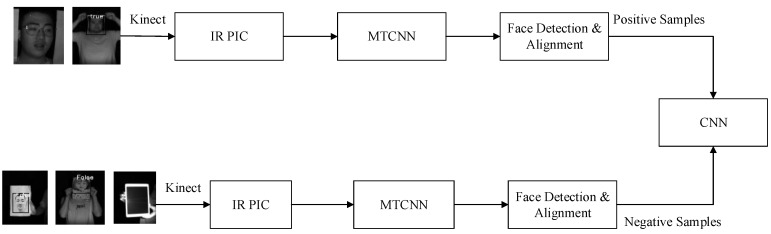
Training process of liveness detection.

Face spoofing detection based on IR images can be treated as a two-classifier issue. This method is used to discriminate fake faces from real ones. The proposed liveness detection algorithm focused on 3D facial space estimation. A Kinect camera was utilized to obtain IR images with deep and gray information. These images were input to the CNN for training to obtain facial skin texture information in space. Because whole facial textures were considered, 2D deceptions, such as those using photos and videos, no longer have any effects. The process of liveness detection based on IR images is shown in Figure 2. IR images of real faces and framed real faces were taken as positive samples, while photos, photos stuck to human faces, and photos on an electronic pad were taken as negative samples after Kinect collection and inputted to the CNN for training.

**Figure 3:**
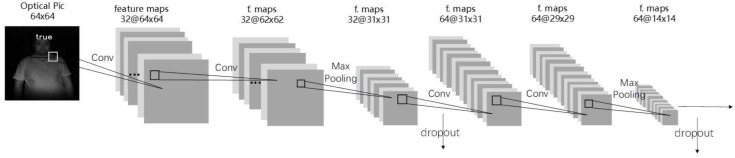
CNN structure.

In this research, a CNN network with four-layer convolution was designed for liveness detection. The CNN structure is shown in Figure 3.

After convolution of the second and fourth layer, a 2 × 2 maximum pooling was adopted. The optimization of dropout at 0.25 was performed after pooling, and optimization of dropout at 0.5 was performed after the first full connection layer. The activation function in the network was relu, and the learning rate was lr = 0.01. The Stochastic Gradient Descent SGD with a momentum of 0.9 were used as optimizers, and the loss function was cross entropy.

First, IR images collected using Kinect were resized to 64 × 64, normalized, and inputted into the network for training. When a new user was added, 10 rounds of training were automatically performed (nb_epoch = 10). As the output category results only included true and false, the “binary-cross threshold” classification function was adopted, and the trained model outputted true or false at the prediction stage. When the output result is true, it means the input image is live, and face recognition is continued using FaceNet; otherwise, spoof information is given, and face recognition is no longer performed.

### 3.4. Improved FaceNet Model for Application-IFaceNet

#### 3.4.1. FaceNet Model

**Figure 4:**
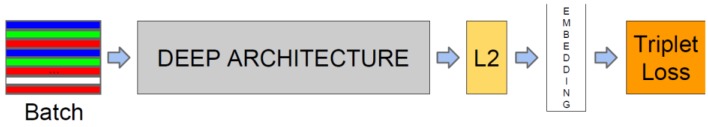
FaceNet structure.

FaceNet consists of a batch input layer and a deep CNN followed by L2 normalization, which results in face embedding. This step is followed by triplet loss during training [2], as shown in Figure 4.

**Figure 5:**
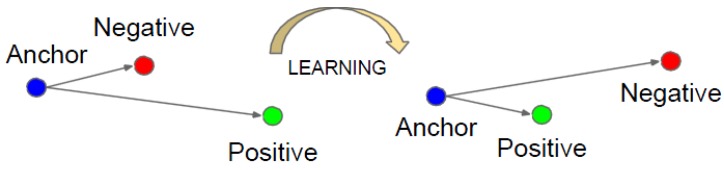
Function of the triplet loss.

FaceNet strives to embed f(x), from image x into a feature space Rd, such that the squared distance between all faces of the same identity is small regardless of imaging conditions, whereas the squared distance between a pair of face images from different identities is large. Triplet loss of the model is shown in Figure 5. The model can minimize the distance between an anchor and a positive of the same identity and maximize the distance between the anchor and a negative of a different identity.

When training FaceNet, three face images, namely X_i_(a), X_i_(p), and X_i_(n), were extracted from the training set each time. These images belonged to anchor, positive, and negative classes. The three pictures formed a triplet. The training network enabled ||f(X_i_(a)) − f(X_i_(p))||^2^ to be as small as possible and the distances ||f(X_i_(a)) − f(X_i_(n))||^2^ and ||f(X_i_(p)) − f(X_i_(n))||^2^ to be as large as possible.

That is, the triple loss satisfies the following equation:

||f(X_i_(a)) − f(X_i_(p))||^2^ + α < ||f(X_i_(a)) − f(X_i_(n))||^2^, (1)

where α is a real number, which makes the distance between facial image features of the same person smaller than the distance between facial features of different people by α. Thus, the loss function is designed as

Loss = ||f(X_i_(a)) − f(X_i_(p))||^2^ + α - ||f(X_i_(a)) − f(X_i_(n))||^2^. (2)

The triplet loss tries to enforce a margin between each pair of faces from one person to all other faces. This procedure allows the faces of one identity to live on a manifold while still enforcing the distance and, thus, the discriminability from other identities. Therefore, the FaceNet model is robust to pose, illumination, and expression [25], which were previously considered to be difficult for face verification systems.

#### 3.4.2. IFaceNet

The original FaceNet model was trained on a labeled faces in the wild (LFW) dataset. In the recognition stage, the trained model extracted the feature vector, which was compared with the faces that have been classified by SVM in the database. A set of predicted values of similarity between the recognized face and various other faces was available in the database, and the maximum of similarity was selected as the output result. If the person is in the database, then the correct identification will be given. However, when the recognized person is not in the database, the classifier will select the person category with the largest predicted value for the output, which results in false identification. Accordingly, we improved the original FaceNet model in two ways for real applications and called it IFaceNet.

##### Set a Valid Threshold

**Figure 6:**
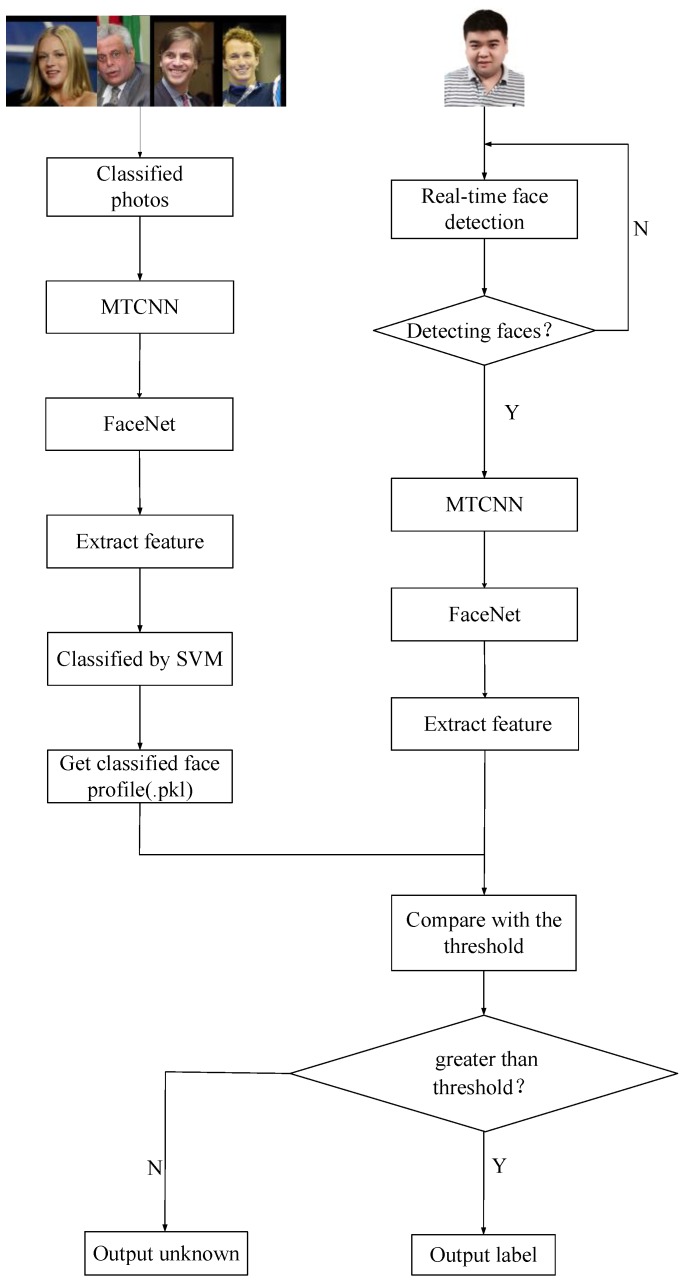
Face recognition process by IFaceNet.

The person is not in the dataset when the recognizing similarity is low. Therefore, we set a threshold of 0.9, and the person was marked as unknown when the predicted maximum was less than 0.9. The modified FaceNet model is shown in Figure 6. By setting the valid threshold to 0.9, unknown people will be filtered effectively so as to reduce the false recognition rate.

##### Building a Unknown Category

We added an “unknown” category and placed a large number of photos from LFW into a named “unknown” folder to reduce the false recognition rate. The unknown folder consisted of approximately 6000 faces from more than 4000 people of LFW. The number of photos in each labelled person should be approximately equal to the number of photos in the unknown folder. When a face is not in the real application dataset, it will have maximum similarity with the unknown category, and an “unknown” will be the output. By adding the unknown category, the original FaceNet model could deal with this case. Thus, we did not need to modify the FaceNet model.

The experimental results showed that both of the improved approaches could reduce the false recognition rate of strangers.

## 4. Experimental Results and Analysis

### 4.1. Experiment Settings

Our model used the Keras framework, which is based on the Tensorflow software library. The hardware platform was an Intel^®^ Core™ i5-8400 with 2.8 GHz, 6-core CPU, 64 GRAM, nvidia GeForce GTX 1080ti, and Ubuntu 18.04.1 OS. The learning rate was set to 0.01, and the decay was set to 1 × 10^−6^. The batch size was 10, and the stochastic gradient descent with momentum optimizer was adopted.

### 4.2. Datasets

**Figure 7:**
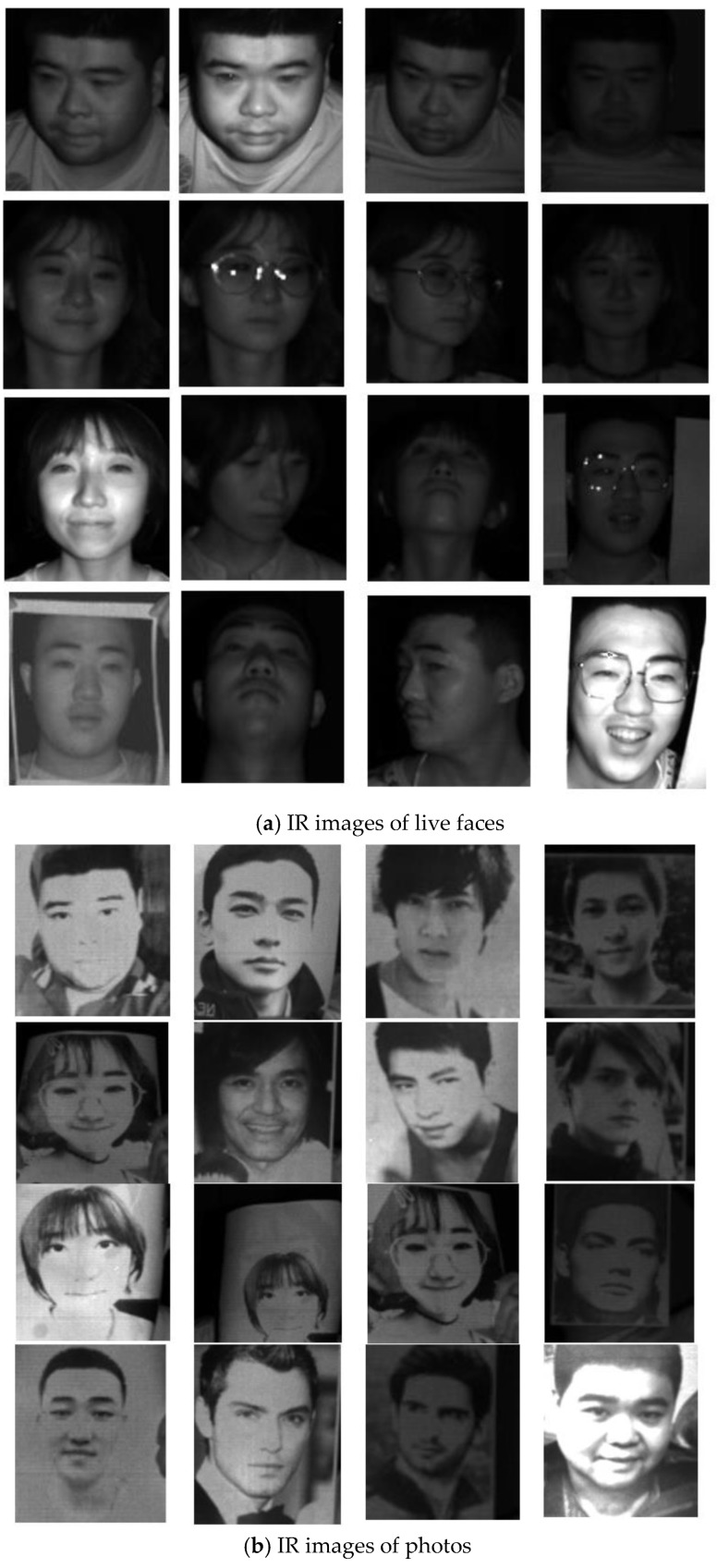
IR pictures collected with a Kinect camera.

The proposed liveness detection algorithm was based on Kinect sensor hardware, and the dataset called NenuLD was our own. NenuLD included 18,036 face photos collected by a Kinect camera. The dataset consisted of a living set and a spoof set. In accordance with common attack forms, the spoof set consisted of photos, photos stuck to human faces, and photos on an electronic pad. The NenuLD dataset is shown in Table 1. The dataset was divided into training, verification, and test sets in the ratio of 8:1:1. IR images acquired using Kinect are shown in Figure 7. Figure 7a presents IR images of a live face collected from a real person, and Figure 7b presents spoof images from photos including some movie stars and ourselves.

**Figure 8:**
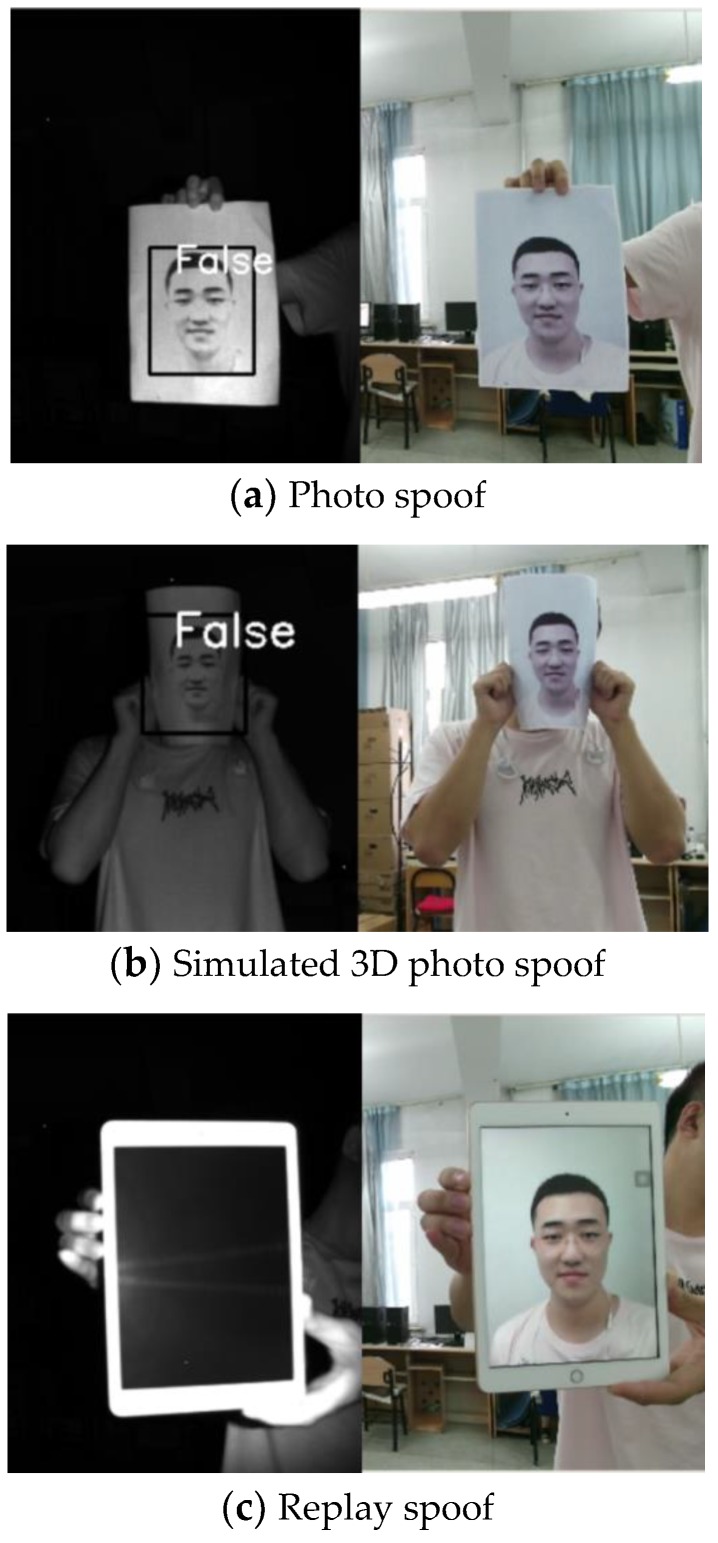
Spoof pictures.

Spoof data are shown in Figure 8. Figure 8a–c show a hand-held photo, a photo stuck to a human face to simulate 3D human face spoofing, and a photo or video on an electronic pad, respectively. The left side presented IR image outputs using Kinect, while the right side presented RGB images. The IR image corresponding to the photo had a clear face contour. The MTCNN could detect and frame the face, and the algorithm recognized the face as false. However, the photo on the electronic pad corresponded to an IR image, which was black and could not be used to detect the outline of the human face; thus, it did not have spoof capability.

**Figure 9:**
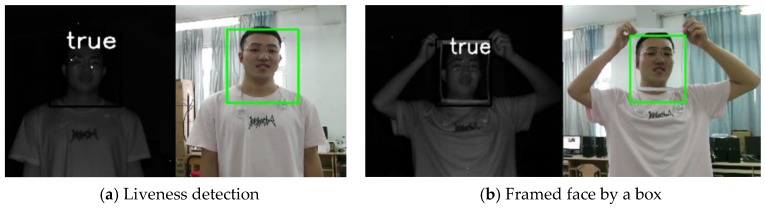
Positive samples.

We also collected framed faces as positive samples, as shown in Figure 9a,b, apart from other real face photos to remove the influence of the photo boundary on liveness detection.

### 4.3. Integrated Experiments

**Figure 10:**
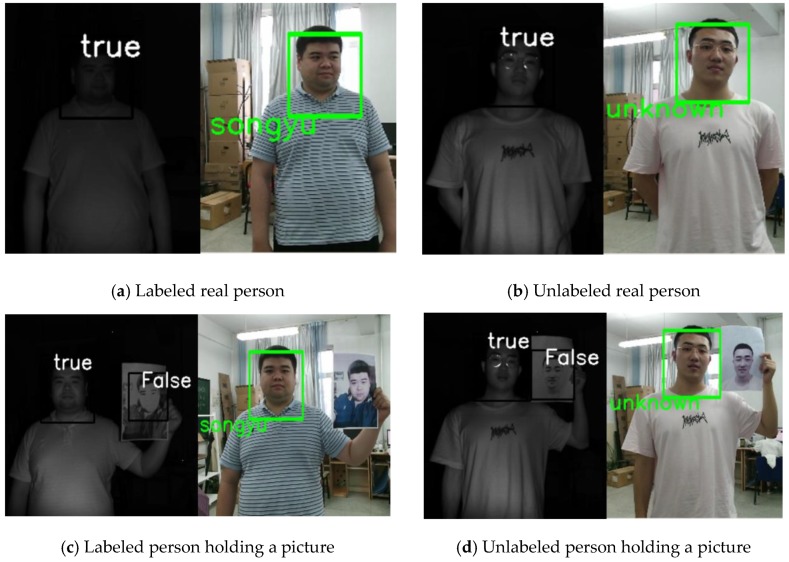

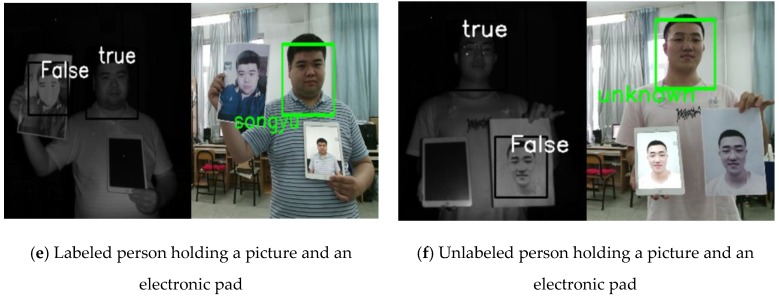
Integrated test.

In this study, the liveness detection algorithm was combined with the face recognition algorithm based on IFaceNet for identity authentication. When the output of the liveness detection algorithm is true, face recognition will be carried out; otherwise, face recognition will not be performed. If the recognized person is in the dataset, then the corresponding label (name) is given; otherwise, “unknown” is displayed. The recognition results are shown in Figure 10.

Figure 10a shows the results for a real person who was labeled in the dataset. Thus, the identity was recognized, and the name of this person was the output. Figure 10b presents the results for a real person who was not in the dataset. Thus, “unknown” was displayed. Figure 10c,d show a hand-held photo, and Figure 10e,f show a hand-held photo, a real person, and a photo on an electronic pad.

### 4.4. Performance Evaluation

The proposed identity authentication algorithm consisted of two parts, namely, liveness detection and face recognition. The liveness detection algorithm consisted of a lightweight CNN model. After the dataset was divided into training, verification, and test sets in the ratio of 8:1:1, the accuracy of the algorithm was 99.8% with our dataset NenuLD. The comparisons with other liveness detection cross-databases are shown in Table 2. Our error recognition accuracy was only 0.2% and higher than others. Because we collected images with a Kinect camera, we could not perform intradatabase comparisons. The improved FaceNet model was trained on the LFW dataset followed by cross-validation. The recognition accuracy was 99.7%. Contrasts between FaceNet and IFaceNet are shown in Table 3. For strangers, IFaceNet had a more accurate recognition output than FaceNet. The whole performance evaluation of the identity authentication algorithm is shown in Table 4. The accuracy of live detection was 99.8% on the NenuLD dataset, and the accuracy of IFaceNet was 99.7%. Thus, the accuracy of the whole identity authentication algorithm was 99.5%, which is equal to the multiplication of both recognition accuracy factors.

The integrated identity authentication algorithm had a time performance of 0.01 s in identifying a photo. Thus, this algorithm can be used for real-time detection and recognition. The IR images for liveness detection were collected using a Kinect camera and, therefore, cannot be compared with the existing face spoof datasets. However, the recognition accuracy from Table 4 indicates that the proposed algorithm was applicable in terms of accuracy and real-time efficiency.

## 5. Conclusions and Future Work

This paper proposed an identity authentication system combining an improved FaceNet model and a liveness detection method. IR images collected by the Kinect camera have depth information. Therefore, the IR pixels from live images have an evident hierarchical structure, while those from photos or videos do not have this feature. Experimental results showed the proposed liveness detection method had a higher recognition accuracy. After that, we improved the FaceNet model for real applications and combine it with liveness detection. The system could effectively solve 2D deception. The IR image features of live faces are greatly different from those of photos or videos, so liveness detection can be treated as a binary classification problem. Thus, a lightweight CNN was designed to realize accurate liveness recognition. The algorithm had a high time efficiency and can be applied in real time.

In future work, more and diverse liveness samples will be collected, and different types of spoofs will be added to detect the reliability of models and algorithms, especially for spoofs in 3D masks and wax figures.

## Figures and Tables

**Table 1:** Liveness detection dataset, called NenuLD.

	Training Images	Validation Images	Test Images
Real faces	8400	1050	1050
Spoof faces	6030	753	753
total	14,430	1803	1803

**Table 2:** Comparison of liveness detection with cross-databases.

	Replay-Attack ERR (%)	CASIA ERR (%)	NenuLD ERR (%)
DOG(baseline) [26]	-	17.0	-
DLTP [27]	7.13	7.02	-
Deep Learning [15]	6.1	7.3	-
DPCNN [17]	2.9	4.5	-
**LDIR**	-	-	**0.2**

**Table 3:** Comparison of FaceNet and IFaceNet.

	People in Dataset	Strangers
FaceNet	Output correct results with 99.7% recognition rate	Output ID with Maximal similarity (error)
IFaceNet	Output correct results with 99.7% recognition rate	Output “unknown” (correct)

**Table 4:** Accuracy of the proposed algorithm.

Accuracy of Live Detection	Accuracy of Face Recognition	Total Accuracy
99.8%	99.7%	99.5%
